# The Ionotropic P2X4 Receptor has Unique Properties in the Heart by Mediating the Negative Chronotropic Effect of ATP While Increasing the Ventricular Inotropy

**DOI:** 10.3389/fphar.2019.01103

**Published:** 2019-09-24

**Authors:** Bruno Bragança, Sílvia Nogueira-Marques, Fátima Ferreirinha, Ana Patrícia Fontes-Sousa, Paulo Correia-de-Sá

**Affiliations:** ^1^Laboratório de Farmacologia e Neurobiologia, Center for Drug Discovery and Innovative Medicines (MedInUP), Instituto de Ciências Biomédicas de Abel Salazar, Universidade do Porto, Porto, Portugal; ^2^Hospital Pedro Hispano, ULS Matosinhos, Matosinhos, Portugal

**Keywords:** ATP, P2X4 receptor, Na^+^/Ca^2+^ exchanger, sinoartrial node, spontaneously beating atria, paced right ventricle

## Abstract

**Background:** Mounting evidence indicate that reducing the sinoatrial node (SAN) activity may be a useful therapeutic strategy to control of heart failure. Purines, like ATP and its metabolite adenosine, consistently reduce the SAN spontaneous activity leading to negative cardiac chronotropy, with variable effects on the force of myocardial contraction (inotropy). Apart from adenosine A_1_ receptors, the human SAN expresses high levels of ATP-sensitive ionotropic P2X4 receptors (P2X4R), yet their cardiac role is unexplored.

**Methods:** Here, we investigated the activity of P2 purinoceptors on isolated spontaneously beating atria (chronotropy) and on 2 Hz-paced right ventricular (RV, inotropy) strips from Wistar rats.

**Results:** ATP (*pEC* *_50_* = 4.05) and its stable analogue ATPγS (*pEC* *_50_* = 4.69) concentration-dependently reduced atrial chronotropy. Inhibition of ATP breakdown into adenosine by NTPDases with POM-1 failed to modify ATP-induced negative chronotropy. The effect of ATP on atrial rate was attenuated by a broad-spectrum P2 antagonist, PPADS, as well as by 5-BDBD, which selectively blocks the P2X4R subtype; however, no effect was observed upon blocking the A_1_ receptor with DPCPX. The P2X4R positive allosteric modulator, ivermectin, increased the negative chronotropic response of ATP. Likewise, CTP, a P2X agonist that does not generate adenosine, replicated the P2X4R-mediated negative chronotropism of ATP. Inhibition of the Na^+^/Ca^2+^ exchanger (NCX) with KB-R7943 and ORM-10103, but not blockage of the HCN channel with ZD7288, mimicked the effect of the P2X4R blocker, 5-BDBD. In paced RV strips, ATP caused a mild negative inotropic effect, which magnitude was 2 to 3-fold increased by 5-BDBD and KB-R7943. Immunofluorescence confocal microscopy studies confirm that cardiomyocytes of the rat SAN and RV co-express P2X4R and NCX1 proteins.

**Conclusions:** Data suggest that activation of ATP-sensitive P2X4R slows down heart rate by reducing the SAN activity while increasing the magnitude of ventricular contractions. The mechanism underlying the dual effect of ATP in the heart may involve inhibition of intracellular Ca^2+^-extrusion by bolstering NCX function in the reverse mode. Thus, targeting the P2X4R activation may create novel well-tolerated heart-rate lowering drugs with potential benefits in patients with deteriorated ventricular function.

## Introduction

Heart rate is primarily set in the right atria by spontaneous generation of rhythmic actions potentials in the sinoatrial node (SAN) (Boyett et al., 2000). The autonomous activity of SAN cardiomyocytes is orchestrated by activation of several ion channels and regulating proteins, which interplay to generate effective action potentials in a regular time basis (Yaniv et al., 2015; Fabbri et al., 2017). The unstable resting membrane potential characteristic of SAN cardiomyocytes is mainly due to mutual influence of intracellular Ca^2+^ “clocks” and membrane potential oscillations. The unstable resting membrane potential and the spontaneous firing of SAN cardiomyocytes are mainly attributed to “funny” currents carried by hyperpolarization-activated cyclic nucleotide-gated channels (HCN) and by the electrogenic Na^+^/Ca^2+^ exchanger (NCX) functioning in the forward Ca^2+^-extrusion mode (Tsutsui et al., 2018; Vinogradova et al., 2018). NCX operates as an integrator of intracellular Ca^2+^ with the membrane potential, in a way that it is responsible by a slow depolarizing current that drives the diastolic depolarization phase in response to calcium leakage from sarcoplasmatic reticulum and other subsidiary Ca^2+^ stores (Bogdanov et al., 2001; Sanders et al., 2006; Groenke et al., 2013; Herrmann et al., 2013). The NCX dynamically fluctuates between forward and reverse modes, thereby extruding or importing Ca^2+^ to subcellular regions, respectively (Samanta et al., 2018). Impairment of the NCX forward-mode by genetic ablation, pharmacological inhibition or even by simply changing its electrochemical gradient consistently induces bradycardia, supporting NCX as a key element in SAN pacemaker activity (Kurogouchi et al., 2000; Bogdanov et al., 2001; Sanders et al., 2006; Groenke et al., 2013; Herrmann et al., 2013).

The SAN, like other regions of the heart, is under control of countless number of signaling molecules, including adenosine triphosphate (ATP) and its derivatives, namely adenosine (Erlinge and Burnstock, 2008; Mangoni and Nargeot, 2008; Burnstock and Pelleg, 2015). Since the pioneering work of Drury and Szent-Gyorgyi almost a century ago (1929), the protective role of purine nucleotides and nucleosides as retaliatory mediators released in response to hypoxia and stressful stimuli in the vascular system has been expanded to the myocardium (Forrester and Williams, 1977), where purines engage energy-saving negative chronotropic, dromotropic and inotropic actions (Versprille and van Duyn, 1966; Lundberg et al., 1984; Pelleg et al., 1987; Belardinelli et al., 1995). Apart from cellular damage, ATP release is not stochastic but rather a fine regulated process (Lazarowski, 2012), involving (1) electrodiffusional movement through membrane ion channels, including pannexin- and connexin-containing hemichannels; (2) facilitated diffusion by nucleotide-specific ATP-binding cassette (ABC) transporters; and (3) cargo-vesicle trafficking and exocytotic granule secretion (Clarke et al., 2009; Pinheiro et al., 2013a; Pinheiro et al., 2013b; Timoteo et al., 2014).

Once in the extracellular milieu, ATP builds up its effects through activation of P2 purinoceptors, which comprise seven ionotropic (P2X1-7) and eight metabotropic (P2Y_1,2,4,6,11,12,13,14_) receptors. Extracellular ATP can also indirectly activate purinoceptors of the P1 family (A_1_, A_2A_, A_2B_, and A_3_) after its conversion into adenosine by cascade of ectonucleoside triphosphate diphosphohydrolases (NTPDases) (Yegutkin, 2008). Intravenous ATP produces negative chronotropy, as well as several other acute cardiovascular effects that resembles adenosine application, almost neglecting ATP-sensitive receptors as active and functional regulators of the cardiac function (Pelleg and Belhassen, 2010). Apart from a massive presence of adenosine A_1_ receptors (Braganca et al., 2016), the SAN from both rodents and humans expresses several P2 purinoceptor subtypes, including ionotropic P2X4, P2X7, and metabotropic P2Y_1_, P2Y_2_, and P2Y_14_ (Musa et al., 2009), yet their role is still largely unknown. Besides species differences, the type and relative abundance of P2 purinoceptors in the heart varies with location and disease conditions (Musa et al., 2009).

Relatively recent evidence called our attention showing that the highly expressed ionotropic P2X4 in the human SAN may be an important modulator of NCX function (Shen et al., 2014). Activation of ionotropic P2X receptors, including the P2X4, triggers the influx of Na^+^ that is subsequently exchanged by Ca^2+^ *via* the NCX (Jarvis and Khakh, 2009). Using various models of heart failure in rodents, it has been demonstrated that reversion of the NCX function by P2X4 activation may lead to improvements of ventricular performance and clinical outcome (Hu et al., 2001; Mei and Liang, 2001; Yang et al., 2004; Yang et al., 2014; Yang et al., 2015). Indeed, pharmacological inhibition of Na^+^/K^+^-ATPase with digitalis interferes with cardiac NCX function, resulting in both negative chronotropy and positive inotropy, thus explaining the rationale for usage of digitalis-like drugs in the management of patients with tachyarrhythmias and ventricular contractile incompetence (Blomstrom-Lundqvist et al., 2003; Ponikowski et al., 2016).

In this study, we aimed at characterizing the effect of the P2X4 on heart rate using isolated spontaneously beating rat atria strips. Taking into consideration that most currently available negative chronotropic drugs are associated with a negative inotropic impact as a major drawback (Ponikowski et al., 2016), experiments were designed to assess the effect of the P2X4 on the magnitude of paced ventricular contractions.

## Materials and Methods

### Animals

Animals care and experimental procedures were conducted in strict accordance with the recommendations of the European Convention for the Protection of Vertebrate Animals used for Experimental and Other Scientific Purposes (ETS 123), Directive 2010/63/EU and Portuguese rules (DL 113/2013). All experimental protocols involving animals were approved by the competent national authority Direção Geral de Alimentação e Veterinária, and by the ICBAS Animal Ethical Committee (No. 224/2017). All efforts were made to minimize animal suffering and to reduce the number of animals used according to the ARRIVE guidelines. Wistar rats (*Rattus norvegicus*; 250–300 g; Charles River, Barcelona, Spain) of either sex were kept at a constant temperature (21 ºC) and a regular light (06:30–19:30 h) – dark (19:30–06:30 h) cycle, with food and water provided *ad libitium*.

### Isolated Spontaneously Beating Atria

Isolated spontaneously beating atria were prepared using a previously described method (Braganca et al., 2016), with some modifications. In brief, hearts were rapidly excised after decapitation followed by exsanguination (Rodent guillotine, Stoelting 51330), and placed in a physiological solution (Tyrode’s solution) composed of (mM): NaCl 137; KCl 4.7; CaCl_2_ 1.8; MgCl_2_ 1; NaH_2_PO_4_ 0.4; NaHCO_3_ 11.9; glucose 11.2 and gassed with 95% O_2_ + 5% CO_2_ (at pH 7.4). Hearts were allowed to beat freely for a few seconds at room temperature, to empty its blood content. The paired rat atria with the SAN region were dissected out, cleaned of fatty tissues, and suspended in a 14-ml organ bath containing gassed Tyrode’s solution at 37 ºC. Each auricular appendage was tied and connected with thread to the organ bath wall and to an isometric force transducer (MLT050/D; AD Instruments, Colorado Springs, CO, USA). Changes in isometric tension were recorded continuously using a PowerLab data acquisition system (Chart 5, version 4.2; AD Instruments, Colorado Springs, CO, USA). The preparations were allowed to equilibrate for 30–40 min. During this time, the preparations were continuously superfused with Tyrode’s solution (1 ml/min) and the tension was adjusted to 9.8 mN. This procedure allows atria (with intact SA node) to progressively recover rhythmic spontaneous beatings (average of 247 ± 5 beats min^−1^ at the beginning of the experimental protocol, n = 79); preparations with spontaneous atrial rate below 200 beats min^−1^ or exhibiting rhythm variations above 10 beats min^−1^ during equilibrium were discarded to ensure measurements were made in atria with intact primary pacemaker SAN activity. None of the preparations exhibited noticeable signs of ectopic-activity caused by secondary pacemakers, usually related to asynchronous and abnormal contractions.

Under these experimental conditions, spontaneously beating rat atria respond to muscarinic and β-adrenergic stimulation, but are unaffected by the application of atropine or atenolol alone used in concentrations high enough (1 µM and 3 µM, respectively) to prevent the effects of acetylcholine (100 µM) and isoproterenol (30 nM), respectively (data not shown). Thus, myographic recordings reported in this study include rate (chronotropic effect) and contractile force (inotropic effect) of spontaneously beating atria measured in the absence of cholinergic and/or adrenergic tone.

### Isolated Paced Right Ventricle Strips

Following the isolation procedures described above for spontaneously beating atria, we also obtained right ventricular (RV) strips (2 mm wide, 8–10 mm long and 1.5 mm thick) by cutting RV-free wall longitudinally to its surface. A pair of ventricular strips was used from each right ventricle. RV strip ends were tied and connected with thread to the 14-ml organ bath hook and to an isometric force transducer (MLT050/D; AD Instruments, Colorado Springs, CO, USA). Changes in the isometric tension of RV strips, measured both by the active tension (mN/mg of wet tissue weight) and by the derivative of developed force over time (+dF/dt, mN/s), were tested at a fixed frequency of 120 beats per min commanded by electric field stimulation of the preparations, so that inotropy was measured without being affected by concurrent changes in chronotropy. Electric pacing (2 Hz, +50% voltage above threshold, 2 ms) was generated by independent Grass S48 stimulators (Quincy, MA, USA) and delivered *via* two platinum electrodes positioned on each side of the preparations. Equilibrium of the preparations was performed as described above for spontaneously beating atria. Only ventricular preparations exhibiting rhythmic contractions with similar amplitude were used.

### Experimental Design

After reaching a steady-state, the Tyrode’s solution flow through the organ bath was stopped and the preparations were incubated for an additional period of 15 min before drug applications. The concentration–response curves for ATP and related nucleotides were performed by non-cumulative application of increasing concentrations of the nucleotides during 5 min followed by a washout period with Tyrode’s solution (15 ml/min) to avoid biases resulting from accumulation of their metabolites and to prevent receptors desensitization. To shorten the experimental duration and, thus, to increase results reproducibility, the majority of the preparations were incubated for 5 min with a fixed concentration (near the EC_50_ value) of the nucleotide either in the absence and in the presence of drug modulator (e.g. receptor antagonist, channel inhibitor); the latter contacted with the preparations at least for 15 min before application of the nucleotide and we kept 2-h washout intervals between testing again the same nucleotide to exclude biases related to P2 purinoceptors desensitization (controls not shown). To avoid damage of RV strips performance by prolonged pacing at 2 Hz, parallel experiments were performed using the two strips coming from the same animal to test the effect of ATP in the absence and in the presence of any modulator.

### Immunofluorescence Staining and Confocal Microscopy Studies

Rat hearts were excised (see above) and placed in oxygenated Tyrode’s solution at 33–34ºC. Following heart excision, the right atrium (RA) containing the SAN region and RV were accurately isolated through the interauricular and interventricular septa and cleaned from tissue debris. Tissue fragments were placed over a small lung lobule fragment with the endocardial layer facing down, stretched to all directions, pinned flat onto cork slices and embedded in Shandon cryomatrix (Thermo Scientific) before frozen in a liquid nitrogen–isopentane; frozen samples were stored at −80ºC until use. Frozen sections with 8 µm thickness were cut perpendicular to the crista terminalis of the RA and parallel to the long axis in the case of RV (see Braganca et al., 2016). Following fixation, the preparations were washed three times for 10 min each using 0.1 M PBS and incubated with a blocking buffer, consisting in fetal bovine serum 10%, bovine serum albumin 1%, Triton X-100 0.3% in PBS, for 2 h. After blocking and permeabilization, samples were incubated with selected primary antibodies (**Table 1**) diluted in incubation buffer (fetal bovine serum 5%, serum albumin 1%, Triton X-100 0.3% in PBS), overnight at 4ºC. For double immunostaining, antibodies were combined before application to tissue samples. Following the washout of primary antibodies with PBS (3 cycles of 10 min) tissue samples were incubated with species-specific secondary antibodies (**Table 1**) in the dark for 2 h, at room temperature. Finally, VectaShield mounting medium with 4′-6-diamidino-2-phenylindole (DAPI) to stain the nuclei (H-1200; Vector Labs) was used, before cover-slipping the glass slides. Observations were performed and analyzed with a laser-scanning confocal microscope (Olympus Fluo View, FV1000, Tokyo, Japan).

**Table 1:** List of primary and secondary antibodies used in immunohistochemistry experiments.

Antigen	Code	Host	Dilution	Supplier
Primary antibodies				
Cx43	Ab11370	Rabbit (rb)	1:700	Abcam
NF-160	Ab7794	Mouse (ms)	1:1,000	Abcam
HCN4	Agp-004	Guinea-pig (gp)	1:150	Alomone
P2X4 (C-terminus)	Apr-002	Rabbit (rb)	1:200	Alomone
P2X4 (extracell. loop)	Apr-024	Rabbit (rb)	1:200	Alomone
NCX1	Anx-011	Rabbit (rb)	1:50	Alomone
Secondary antibodies				
Alexa Fluor 488 anti-rb	A-21206	Donkey	1:1,500	Molecular probes
Alexa Fluor 568 anti-ms	A-10037	Donkey	1:1,500	Molecular probes
TRITC 568 anti-gp	706-025-148	Donkey	1:150	Jackson Immuno Res.

The SAN was characterized as described in a previous study from our group (Braganca et al., 2016). SAN identification was also facilitated by observation of the sinus node artery surrounded by small-size cardiomyocytes positive for the hyperpolarization-activated cyclic nucleotide-gated channel 4 (HCN4) and negative against connexin-43 (Cx43), a gap junction protein ubiquitously expressed in the heart apart from in nodal tissue. In some of the experiments, the existence of a large number of neurofilament 160 (NF-160) positive neuronal fibers was also used to identify the SAN region (Tellez et al., 2006; Braganca et al., 2016).

### Solutions and Chemicals

Adenosine-5′-triphosphate (ATP); adenosine 5′-[γ-thio]-triphosphate (ATPγS); cytidine-5′-triphosphate (CTP); 1,3-dipropyl-8-cyclopentyl-xanthine (DPCPX); 22,23-dihydroavermectin B1 (ivermectin); and 2-[(3,4-dihydro-2-phenyl-2H-1-benzopyran-6-yl)oxy]-5-nitro-pyridine (ORM-10103) were obtained from Sigma (Poole, U.K.). 5-(3-bromophenyl)-1,3-dihydro-2H-benzofuro[3,2-e]-1,4-diazepin-2-one (5-BDBD); 3-[[5-(2,3-dichlorophenyl)-1H-tetrazol-1-yl]methyl]pyridine hydrochloride (A438079); 2-[2-[4-(4-nitrobenzyloxy)phenyl]ethyl]isothiourea mesylate (KR-R7943) and sodium metatungstate (POM-1) were from Tocris Cookson Inc. (Bristol, UK). Pyridoxalphosphate-6-azophenyl-2′,4′-disulphonic acid (PPADS) and ZD7288 were from Ascent Scientific (Bristol, UK). Dimethylsulfoxide (DMSO), serum albumin and Triton X-100 were from Merck (Darmstadt, Germany). DPCPX was made up in 99% DMSO/1% NaOH 1 mM (v/v); 5-BDBD, A438079, Ivermectin, ORM-10103, KB-R7943 were made up in DMSO. Other drugs were prepared in distilled water. All stock solutions were stored as frozen aliquots at −20°C. Dilutions of these stock solutions were made daily and kept protected from the light to prevent photodecomposition. No statistically significant differences between control experiments, made in the absence or in the presence of the solvents at the maximal concentrations used (0.5% v/v), were observed. The pH of the Tyrode’s solution did not change by the addition of the drugs in the maximum concentrations applied to the preparations.

### Presentation of Data and Statistical Analysis

The isometric contractions were recorded and analyzed before and after the addition of each drug at the desired concentration. Results were presented as percentages of variation compared to baseline (% Δ baseline), obtained before application of the test drug. Data are expressed as mean ± SEM, with *n* indicating the number of animals used for a particular group of experiments. Graphical data are expressed as box-and-whiskers plots, with whiskers ranging from minimum to maximum values calculated as a percentage of variation from baseline. Since modulation of ATP-mediated responses were unpredictable, we did not apply any power calculations to pre-determine sample size, thus we pre-specified a number of 4–7 experiments for each condition. Concentration–response curves were analyzed by fitting four-parameter logistic sigmoidal functions to the experimental data to estimate pEC_50_ for negative chronotropy and inotropy of the nucleotides. All curve fitting procedures, graphical, and statistical analyses were carried out using GraphPad Prism 7.04 for Windows software (La Jolla, USA). Spontaneous or electrically-evoked mechanical tension (inotropic effect) and contraction rate (chronotropic effect) were evaluated using the Student’s *t*-test for paired samples assuming a Gaussian distribution of data. Given that significant variability (ranging from 15 to 35%) was observed amongst animals for the negative chronotropic action of ATP (100 µM), changes on the nucleotide effect in the presence of a modulator was always compared to its absence in the same animal or preparation to make differences between paired values consistent. A value of *p* < 0.05 was considered to represent a significant difference.

## Results

### Effects of ATP on Sinoatrial Chronotropy and Right Ventricular Inotropy

**Figure 1:**
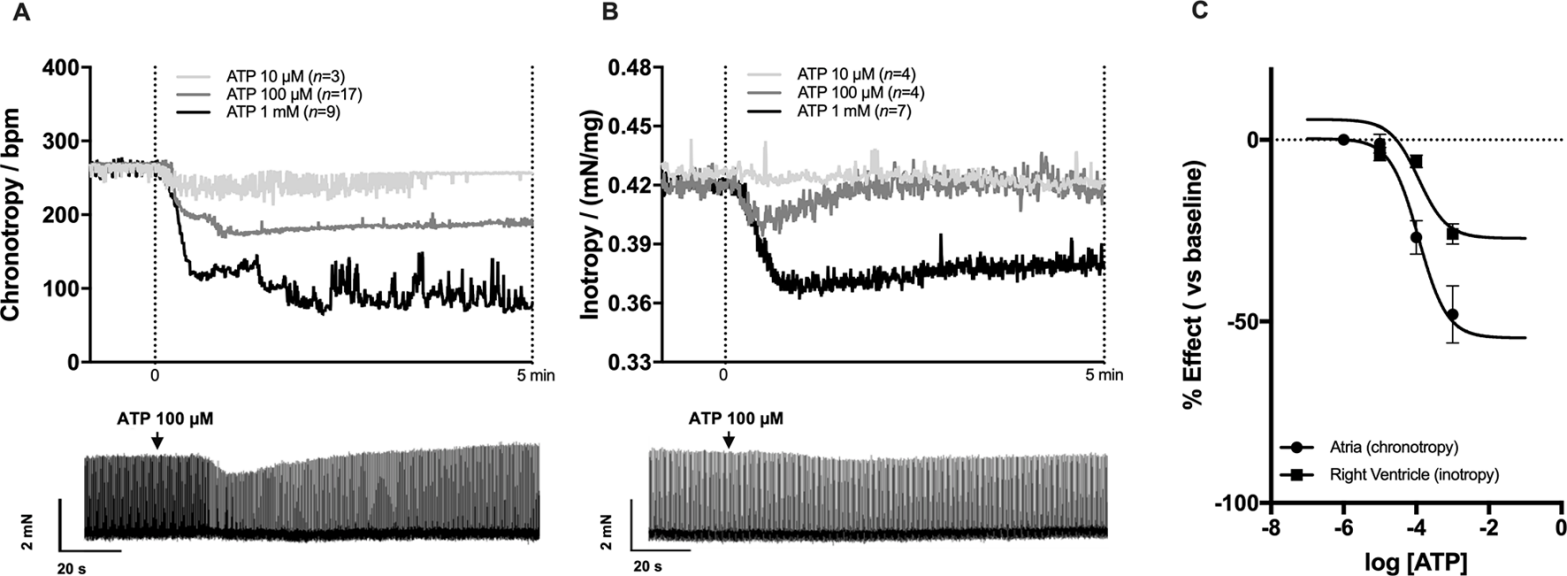
Effects of ATP on spontaneously beating atria (**A**, chronotropy, beats min^−1^) and 2 Hz-paced right ventricular (**B**, inotropy, mN/mg of tissue) strips from Wistar rats. ATP (0.01–1 mM) was applied non-cumulatively for 5 min followed by a washout period to avoid biases resulting from bath accumulation of metabolites and from receptors desensitization. Upper panels **(A)** and **(B)** show average values; bottom panels show typical myographical recordings of atrial and ventricular preparations challenged with ATP (100 µM). Panel **(C)** shows the concentration-response curves for ATP (0.001–1 mM) fitted by four-parameter logistic sigmoidal functions used to estimate pEC_50_ values for the negative chronotropic and inotropic effects of the nucleotide. Data are expressed as mean ± SEM from an *n* number of animals indicated in upper panels **(A)** and **(B)**, respectively.

Non-cumulative application of ATP (0.001–1 mM) concentration-dependently decreased atrial chronotropy and right ventricular inotropy (**Figure 1**). The onset of ATP response was readily visible in about 30 s for both myocardial preparations; it reached a sustained maximal effect roughly 1 min after application and lasted while the nucleotide was kept in the incubation fluid, *i.e.* at least for 5 min. Spontaneously beating atria were slightly more sensitive (*p* < 0.05) to the inhibitory effect of ATP compared to paced RV strips (**Figure 1C**). The estimated pEC_50_ for the negative chronotropic and inotropic ATP responses were 4.05 and 3.45, respectively. Of note, ATP exhibited a biphasic effect on atrial inotropy, which was characterized by an initial decrease in the magnitude of atrial contractions followed by a gradual recovery to levels above the baseline (**Figure 1A**), as reported by other authors (Froldi et al., 1994; Gergs et al., 2008).

### The Negative Chronotropic Effect of ATP Depends of P2 Purinoceptors Activation

**Figure 2:**
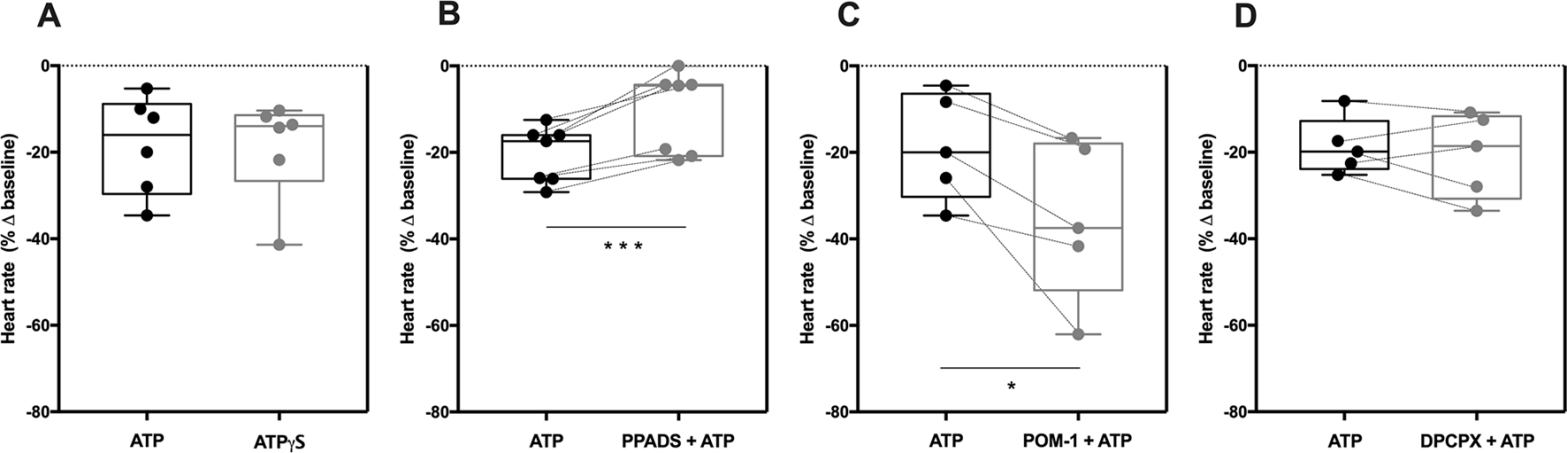
The negative chronotropic effect of ATP depends of P2 purinoceptors activation. The negative chronotropic effect of ATP (100 µM) was tested either in the absence or in the presence of the non-selective P2 receptor antagonist, PPADS (10 µM, **B**), the NTPDase inhibitor, POM-1 (100 µM, **C**), and the selective adenosine A_1_ receptor antagonist, DPCPX (3 nM, **D**). The negative chronotropic effect of the enzymatically stable ATP analogue, ATPγS (100 µM, **A**), is also shown for comparison. Represented are box-and-whiskers plots, with whiskers ranging from minimum to maximum values calculated as a percentage (%) of variation from baseline; horizontal lines inside boxes indicate the corresponding medians. Each data point represents the result of a single experiment; data from the same experiment are connected by lines. *p < 0.05, ***p < 0.001 (Student’s *t*-test for paired samples) represent significant differences when compared to the effect of ATP alone.

The negative chronotropic effect of ATP following intravenous application of the nucleotide resembles that obtained after administration of adenosine (Pelleg and Belhassen, 2010), the end product of ATP hydrolysis by the ectonucleotidase cascade (Cardoso et al., 2015). To know whether ATP is acting directly on P2 purinoceptors or indirectly *via* P1 receptors after its extracellular conversion into adenosine, we tested the effect of ATPγS, an enzymatically-stable ATP analogue. ATPγS (100 µM) decreased sinoatrial chronotropy (−19 ± 5%, n = 6) by a similar extent to that observed for ATP (100 µM, −18 ± 5%, n = 6) (**Figure 2A**); the estimated pEC_50_ for the negative chronotropic effect of ATPγS was 4.69 (**Supplementary Figure S1A**). Moreover, the broad-spectrum P2 receptor antagonist PPADS (10 µM) significantly attenuated the negative chronotropic response of ATP (−20 ± 2% vs −11 ± 4%, n = 7, *p* < 0.001) (**Figure 2B**); the blocking effect of PPADS was more evident upon increasing the concentration of the P2 receptor antagonist to 100 µM (−17 ± 4% vs −2 ± 2%, n = 4) (see **Supplementary Figure S1D**).

Blockage of ATP breakdown by NTPDases with POM-1 (100 µM) significantly (*p* < 0.05) potentiated the negative chronotropic response of ATP (100 µM) (−19 ± 6% vs −35 ± 8%, n = 5, *p* < 0.05) (**Figure 2C**), whereas the selective adenosine A_1_ receptor antagonist, DPCPX (3 nM) (Lohse et al., 1987), was ineffective (−19 ± 3% vs −21 ± 4%, n = 5; *p* > 0.05) (**Figure 2D**). Indeed, the NTPDase inhibitor, POM-1 (100 µM), shifted to the left (*pEC* *_50_* = 5.10; *p* < 0.05) the concentration-response curve of ATP (0.001–1 mM) without significantly modifying the Hill slope (see **Supplementary Figure S1B**).

On their own, PPADS (10 µM and 100 µM), POM-1 (100 µM) and DPCPX (3 nM) were virtually devoid of effect on spontaneous atrial beating rate (see **Supplementary Figure S2**). It is also worth noting that blockage of muscarinic acetylcholine receptors with atropine (1 µM) did not modify the negative chronotropic effect of ATP (100 µM) (data not shown), ruling out putative changes in the cholinergic tone operated by ATP.

### The Negative Chronotropic Effect of ATP Is Mediated by P2X4 Receptors Activation

The P2 purinoceptors expression in the SAN is species specific. For instance, in humans the rank order of expression of ionotropic P2X receptors is the following: P2X4 > P2X7> > P2X1 > P2X5 (P2X2 and P2X3 are absent), while in rats it is P2X5> > P2X7> > P2X4∼P2X1∼P2X2 > P2X3 (Musa et al., 2009). Regrettably, there are no specific pharmacologic agonists or antagonists to the P2X5 receptor. It is worth noting that the P2X7 receptor is negatively modulated by extracellular Ca^2+^ and shows low affinity (0.1–1mM) for ATP. This contrasts with the estimated EC_50_ values in the low micromolar range for ATP and ATPγS that is characteristic of the most abundant P2X4 receptor in the human SAN (Soto et al., 1996; Michel et al., 1997). Interestingly, both P2X4 and P2X7 receptor pores are able to translocate extensive amounts of Na^+^ into the cells. These premises prompted us to test whether these receptors could be involved in the negative chronotropic effect of ATP.

**Figure 3:**
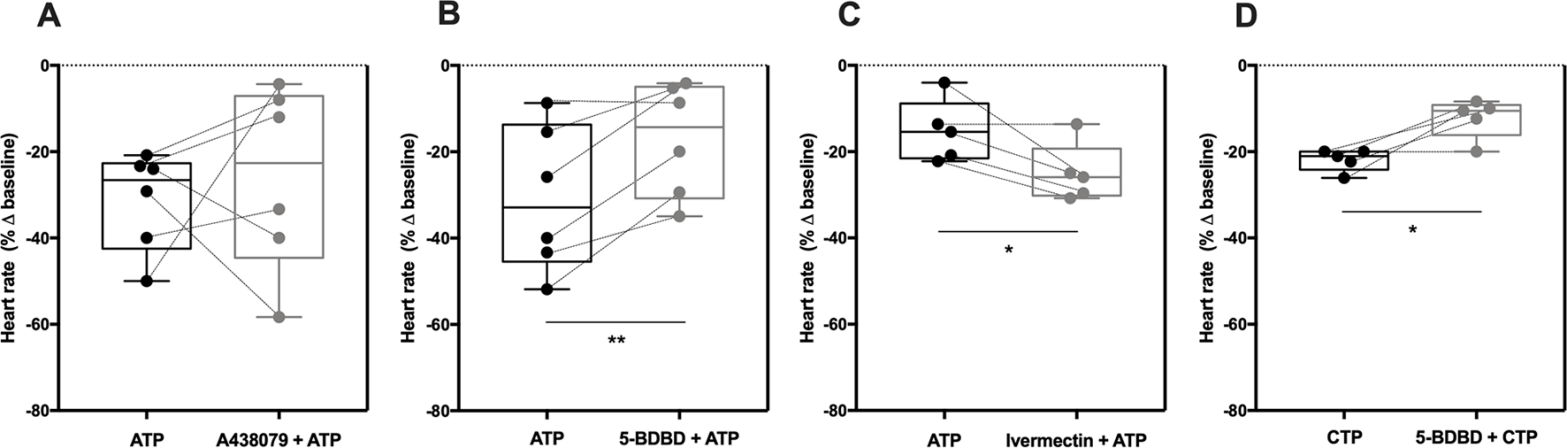
The negative chronotropic effect of ATP is mediated by activation of P2X4. The negative chronotropic effect of ATP (100 µM) was tested either in the absence or in the presence of the P2X7 receptor antagonist, A438079 (3 µM, **A**), the P2X4 receptor antagonist, 5-BDBD (10 µM, **B**) and the positive allosteric modulator of the P2X4 receptor, ivermectin (30 µM, **C**). The negative chronotropic effect of CTP (1 mM, **D**) either in the absence or in the presence of 5-BDBD (10 µM), is also shown for comparison. Represented are box-and-whiskers plots, with whiskers ranging from minimum to maximum values calculated as a percentage (%) of variation from baseline; horizontal lines inside boxes indicate the corresponding medians. Each data point represents the result of a single experiment; data from the same experiment are connected by lines. *p < 0.05, **p < 0.01 (Student’s *t*-test for paired samples) represent significant differences when compared to the effects of ATP or CTP alone, respectively.

Selective blockage of the P2X7 receptor with A438079 (3 µM, **Figure 3A**) failed to modify the negative chronotropy effect of ATP (100 µM) (−31 ± 5% vs −26 ± 9%, n = 6, *p* > 0.05), whereas the potent and selective P2X4 receptor antagonist, 5-BDBD (10 µM) (Coddou et al., 2019), significantly attenuated ATP-induced negative chronotropism (−31 ± 7% vs −17 ± 5%, n = 6, *p* < 0.05) (**Figure 3B**). The negative chronotropic action of ATP (100 µM) was potentiated by ivermectin (30 µM) (−16 ± 3% vs −25 ± 3%, n = 5, *p* < 0.05) (**Figure 3C**), a drug that acts as positive allosteric modulator of the P2X4 receptor *via* a dual mechanism that involves potentiation and delayed inactivation of its currents, exhibiting selectivity over other P2X receptors (Khakh et al., 1999). The concentration-response curve of ATP (0.001–1 mM) was shifted to the left (*pEC* *_50_* = 4.99; *p* < 0.05) by ivermectin (30 µM) compared to the effect of ATP alone (see **Supplementary Figure S1C**). Please note that, on their own, A438079 (3 µM, −5 ± 3%, n = 6), 5-BDBD (10 µM, −8 ± 9%, n = 5) and ivermectin (30 µM, 3 ± 3%, n = 5) were virtually devoid of effect on the spontaneous atrial frequency (see **Supplementary Figure S2**).

In order to further explore the potential involvement of the P2X4 receptor, we used CTP as a preferential P2X receptor agonist whose hydrolysis does not directly generate adenosine or other adenine nucleotides. Despite the fact that the P2X4 receptor exhibits low affinity for CTP compared to ATP (Soto et al., 1996; Kasuya et al., 2017), CTP (1 mM) decreased the spontaneous atrial rate by 22 ± 1% (n = 5) and this effect was also antagonized by 5-BDBD (10 µM, −12 ± 2%, n = 5, *p* < 0.05) (**Figure 3D**).

### The P2X4-Mediated Negative Chronotropic Effect of ATP Involves NCX, But Not HCN

ATP binding to the P2X4 receptor dramatically increases Na^+^ and Ca^2+^ influx through the receptor pore, which may interfere with NCX function as one of the sarcolemma controllers of the SAN pacemaker activity (Shen et al., 2014). Besides NCX, the normal sinus rhythm also depends on HCN channels mediating *I* *_f_* currents (Bogdanov et al., 2001; Sanders et al., 2006; Groenke et al., 2013; Herrmann et al., 2013). Therefore, we thought it was relevant to evaluate the P2X4 receptor influence on downstream activation of NCX and/or HCN membrane transporters, which are essential to control heart rate. To this end, we used two different compounds known to inhibit NCX activity, namely KB-R7943 and the recently developed ORM-10103 (Jost et al., 2013). Since both inhibitors have putative negative chronotropic actions, we performed concentration–response curves to determine the minimal concentration beyond that reduction of chronotropy would be a problem in interaction experiments (data not shown). Incubations of KB-R7943 and ORM-10103 for 15 min at a final concentration of 3 µM had no effect on spontaneously beating atria strips (−3 ± 3%, n = 6, *p* > 0.05 vs baseline; −1 ± 2%, n = 5, *p* > 0.05 vs baseline; respectively) (**Supplementary Figure S2**).

**Figure 4:**
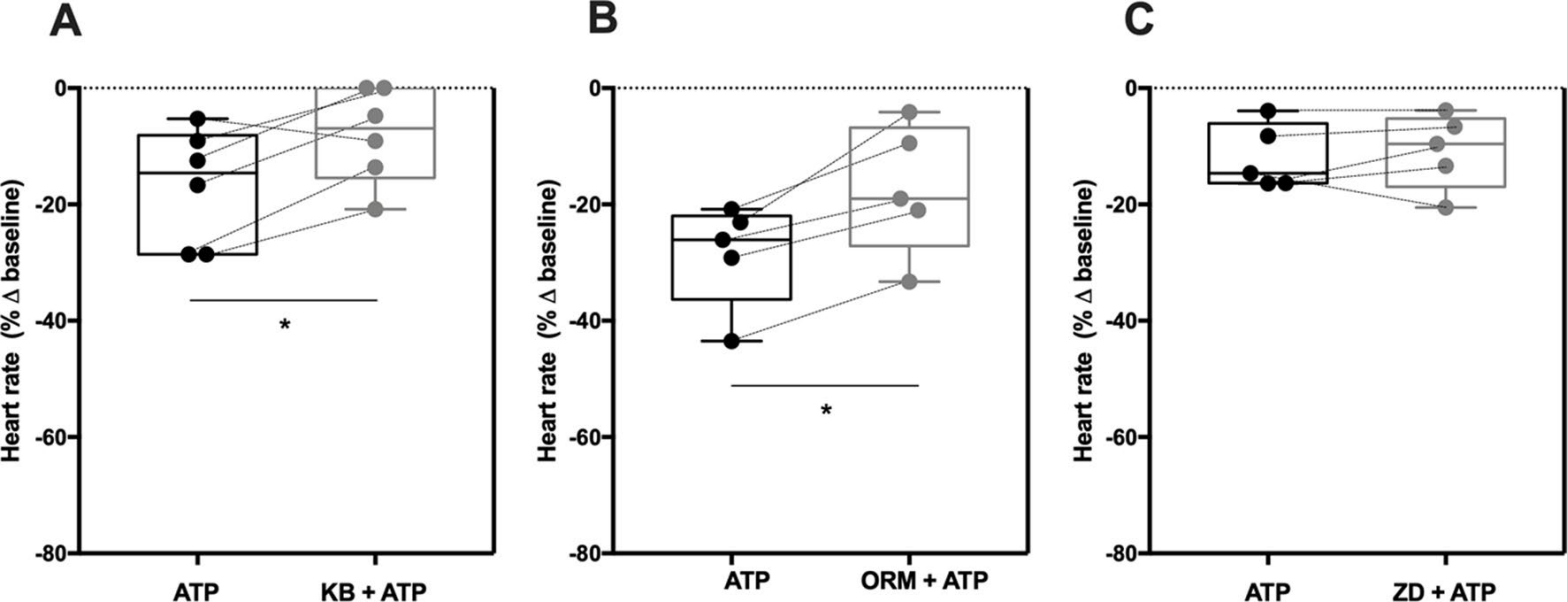
The negative chronotropic effect of ATP involves ion exchange *via* pacemaker NCX, but not HCN. ATP (100 µM)-induced negative chronotropism was tested either in the absence or in the presence of two NCX inhibitors, KB-R7943 (3 µM, **A**) and ORM-10103 (3 µM, **B**), and of a HCN channel inhibitor, ZD7288 (300 nM, **C**). Represented are box-and-whiskers plots, with whiskers ranging from minimum to maximum values calculated as a percentage (%) of variation from baseline; horizontal lines inside boxes indicate the corresponding medians. Each data point represents the result of a single experiment; data from the same experiment are connected by lines. *p < 0.05 (Student’s *t*-test for paired samples) represent significant differences when compared to the effect of ATP alone.

The negative chronotropic effect of ATP (100 µM) was attenuated by KB-R7943 (3 µM; −19 ± 4% vs −8 ± 3%, n = 6, *p* < 0.05) and by ORM-10103 (3 µM; −29 ± 4% vs −17 ± 5%, n = 5, *p* < 0.05) (**Figures 4A, B**); the inhibitory effect of these compounds had a similar magnitude to that observed with the P2X4 antagonist, 5-BDBD (10 µM) (see **Figure 3B**). Pre-incubation with the ivabradine-like HCN channel inhibitor ZD7288 (300 nM) did not significantly modify the chronotropic effect of ATP (100 µM) (−12 ± 2% vs −11 ± 3%, n = 5, *p* > 0.05) (**Figure 4C**). On its own, ZD7288 (300 nM) decreased the spontaneous atrial rate only by 6 ± 2% below the control (n = 5, *p* < 0.05) (**Supplementary Figure S2**).

### P2X4-Induced NCX Transport Reversal Counteracts the Negative Inotropic Effect of ATP in Paced Ventricular Strips

**Figure 5:**
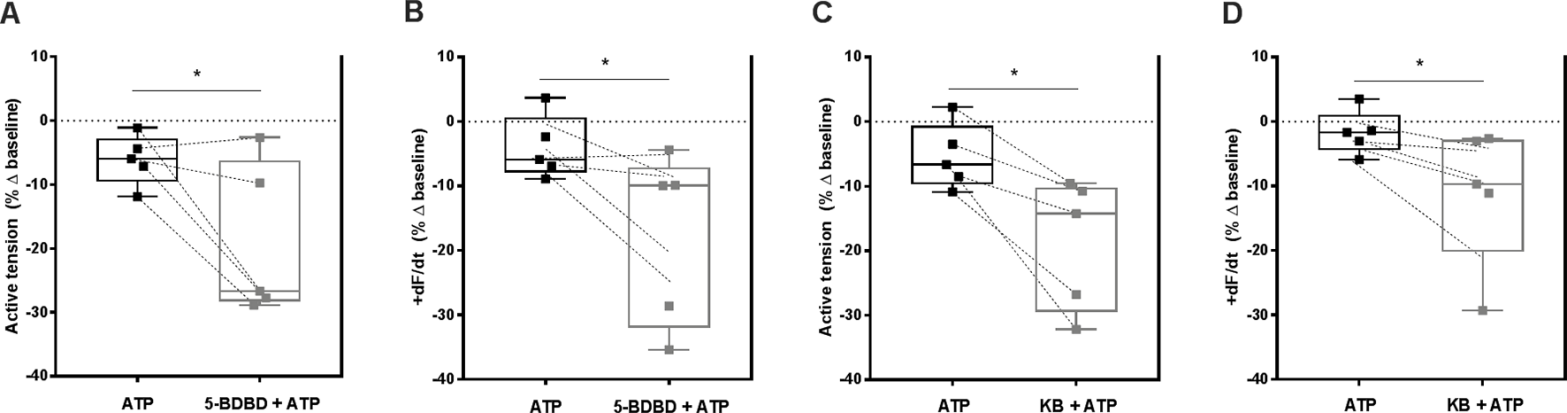
Selective blockage of P2X4 and of NCX transporter partially offsets the negative inotropic effect of ATP in paced rat ventricular strips. The negative inotropic effect of ATP (100 µM) was tested either in the absence or in the presence of the P2X4 receptor antagonist, 5-BDBD (10 µM, **A** and **B**) and of the NCX inhibitor, KB-R7943 (3 µM, **C** and **D**). Represented are box-and-whiskers plots, with whiskers ranging from minimum to maximum values calculated as a percentage (%) of variation from the baseline isometric tension of RV strips, measured as the active tension (mN/mg of wet tissue weight, panels **A** and **C**) and the derivative of developed force over time (+dF/dt, mN/s, panels **B** and **D**); horizontal lines inside boxes indicate the corresponding medians. Each data point represents the result of a single experiment; data from the same experiment are connected by lines. *p < 0.05 (Student’s *t*-test for paired samples) represent significant differences when compared to the effect of ATP alone.

Apart from digitalis, heart rate slowing drugs used in clinical practice decrease cardiac inotropism as a major drawback (Ponikowski et al., 2016). As shown in **Figure 1B**, ATP (0.001–1 mM) concentration-dependently decreased the amplitude of paced RV contractions. **Figure 5** shows that blockage of P2X4 receptors with 5-BDBD (10 µM) augmented the negative inotropic effect of ATP (100 µM) measuring the percent variation of the active tension (−6 ± 2% vs −19 ± 5%, n = 5, *p* < 0.05; **Figure 5A**) or of the derivative of developed force over time (+dF/dt) (−4 ± 2% vs −18 ± 6%, n = 5, *p* < 0.05; **Figure 5B**) in paced RV strips. The effect of 5-BDBD (10 µM) was mimicked by KB-R7943 (3 µM), *i.e.* inhibition of NCX sensitized RV strips to the negative inotropic effect of ATP (100 µM) calculated also measuring the percent variation of the active tension (−5 ± 2% vs −19 ± 5%, n = 5, *p* < 0.05; **Figure 5C**) or of the derivative of developed force over time (+dF/dt) (−2 ± 2% vs −12 ± 4%, n = 5, *p* < 0.05; **Figure 5D**). On their own, 5-BDBD (10 µM) and KB-R7943 (3 µM) marginally reduced ventricular inotropy by 14 ± 4% (n = 5, *p* < 0.05) and by 11 ± 19% (n = 5, *p* > 0.05), respectively (**Supplementary Figure S3**). These findings suggest that activation of the P2X4 partially counteracts the negative inotropic effect of ATP probably by reversing the NCX electrogenic current to pump Na^+^ out and Ca^2+^ into ventricular cardiomyocytes.

### Localization of P2X4, NCX1, and HCN4 Proteins in the Rat Heart

**Figure 6:**
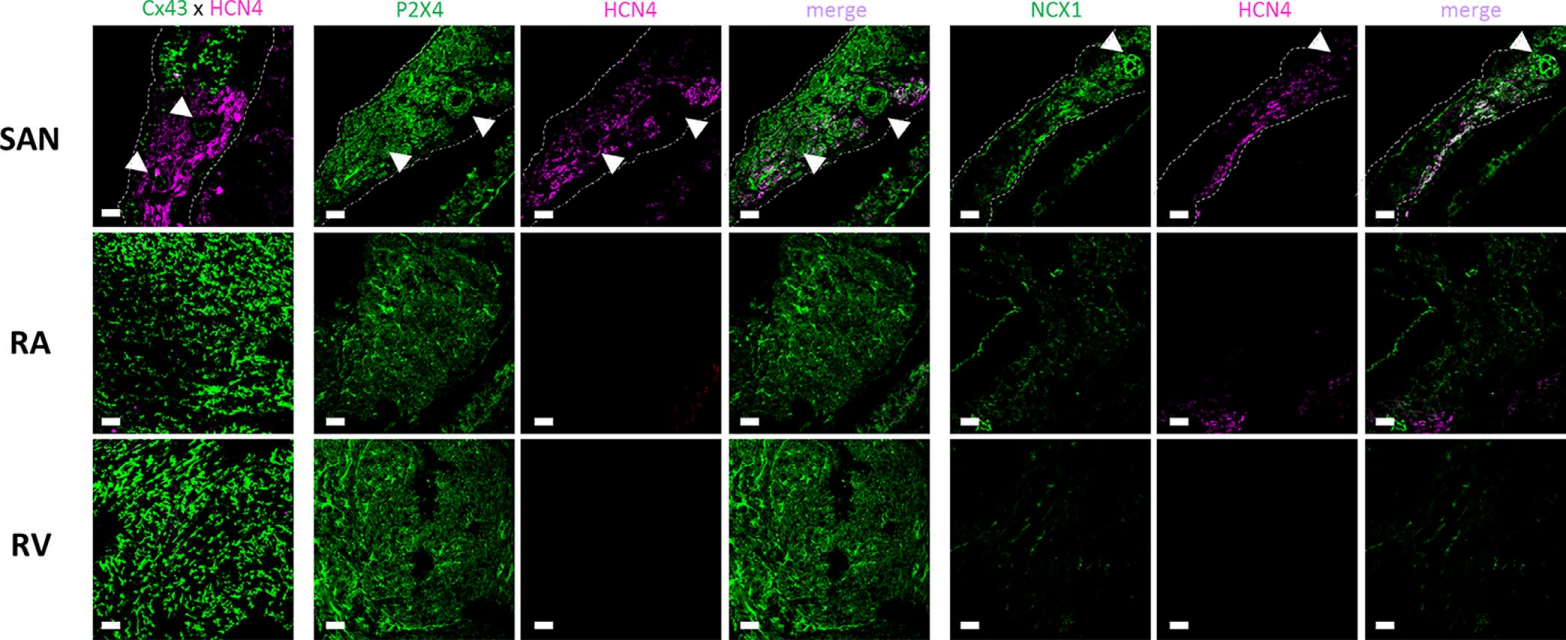
Representative confocal micrographs showing the immunolocalization of the P2X4 receptor (Apr-002, C-terminus, Alomone) and NCX1 (Anx-011, Alomone) protein in the sinoatrial node (SAN), right atria (RA) and right ventricle (RV). The SAN was identified based on its low Cx43 (green) and high HCN4 (magenta) protein expression (left hand-side images). Images were taken from whole-mount heart preparations including the three analyzed regions, SAN, RA and RV. Dashed lines represent boundaries of the SAN. The pulmonary parenchyma was used as a structural support to facilitate immunostaining of myocardial sections and it is visible in the bottom right quadrant of each SAN image. White arrows indicate blood vessels including the SAN artery. Scale bar 30 µm. Images are representative of three different individuals.

Confocal micrographs shown in **Figure 6** demonstrate that P2X4 receptor protein is expressed in the plasma membrane of cardiomyocytes of all assayed regions of the rat heart; in these experiments we used a knock-out validated antibody targeting the amino acid residues 370–388 of the C-terminus of the rat P2X4 receptor (Apr-002 from Alomone). Using tissues prepared in identical conditions and visualized with the same acquisition settings, one may conclude that the P2X4 receptor expression is higher in the SAN followed by the RV and RA. This regional difference was confirmed using a distinct antibody targeting amino acid residues 301–313 of the extracellular loop of the rat P2X4 receptor (Apr-024 from Alomone) (**Supplementary Figure S4**).

The immunoreactivity against NCX1 protein followed the same staining pattern to that found for the P2X4 receptor; the strongest immunofluorescence signal was also found in the SAN followed by other regions of the rat heart (**Figure 6**). Cardiomyocytes of the SAN region staining positively against NCX1 also exhibit immunoreactivity against the HCN4 protein. The same occurred regarding co-localization of P2X4 and HCN4. Taking this into consideration, even though double immunolabelling against P2X4 and NCX1 was not possible because available antibodies were raised in the same species (rabbit), it looks like that the staining pattern obtained with both P2X4 and NCX1 antibodies indicates that they may co-localize in HCN4 positive cardiomyocytes of the SAN (**Figure 6**). Please note that the smooth muscular layer of SAN blood vessels also exhibits strong immunoreactivity against P2X4 and NCX proteins (**Figure 6**, arrow heads). Likewise, these two proteins also co-localize with neurofilament 160 (NF160) in neuronal fibers of the SAN region (**Supplementary Figure S5**).

## Discussion

**Figure 7:**
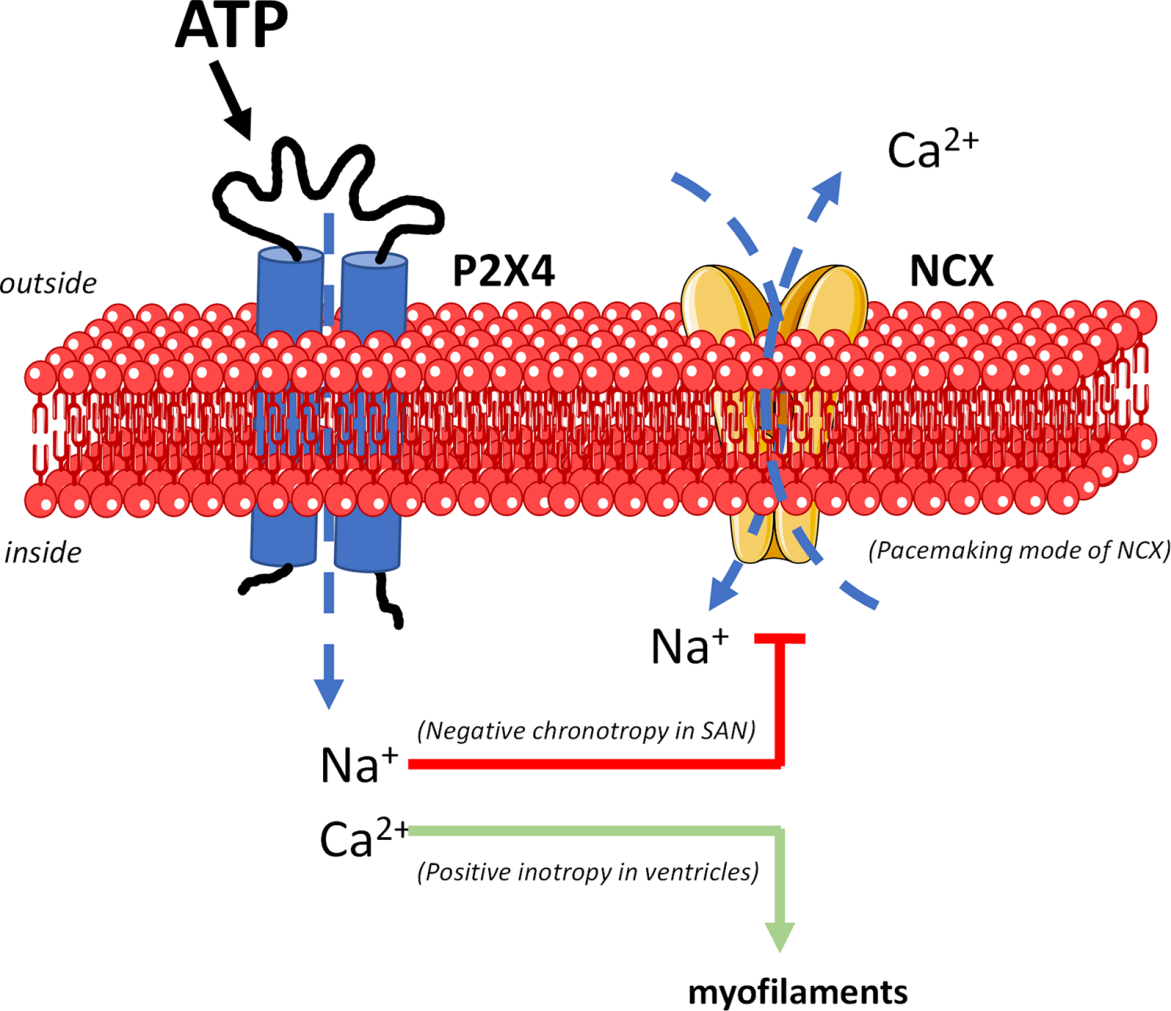
The mechanism underlying the dual P2X4 receptor-mediated effects on cardiac chronotropy and inotropy implicates downstream modulation of NCX activity (digitalis-like phenomenon). Besides ion fluxes carried by pacemaker HCN channels (not represented), the unstable resting membrane potential and the spontaneous firing of SAN cardiomyocytes are attributed mainly to the electrogenic NCX transport operating in the forward Ca^2+^-extrusion mode. Na^+^ influx through the P2X4 receptor pore dissipates the electrochemical gradient of this ion across the plasma membrane leading to inhibition and/or reversion of the NCX pacemaker current. This may justify slowing down of SAN cells depolarizations and the negative chronotropic effect of ATP. Likewise, intracellular Ca^2+^ accumulation due both (1) to Ca^2+^ influx through the P2X4 receptor pore, and (2) to reversal of NCX activity may explain the positive inotropic effect of the P2X4 receptor in paced ventricular cardiomyocytes. Figure composition used elements from *Servier Medical Art*.

Data suggest that activation of ATP-sensitive P2X4 receptors plays a major contribution in decreasing the spontaneous activity of the SAN while partially offsetting the negative inotropic effect of the nucleotide by downstream reversing the electrogenic NCX mode of function (**Figure 7**).

### ATP-Sensitive P2X4 Receptors Decrease Sinoatrial Pacemaker Activity

Extracellular ATP is an endogenous regulator of the cardiovascular function by acting either directly on P2 receptors or indirectly on P1 receptors after its breakdown to adenosine by NTPDases (Yegutkin, 2008; Headrick et al., 2013; Burnstock and Pelleg, 2015). Although the mammalian myocardium expresses multiple purinoceptors (Musa et al., 2009), the adenosine A_1_ receptor has received most attention due to its relative abundance and well characterized functional role in the acute regulation of the heart (Musa et al., 2009; Chandrasekera et al., 2010; Headrick et al., 2013). Adenosine A_1_ receptors activation decreases cardiac chronotropy, dromotropy, inotropy and counteracts adrenergic stimulation by a dual mechanism involving inhibition of adenylyl cyclase and opening of potassium channels (Belardinelli and Lerman, 1991; Burnstock and Pelleg, 2015; Braganca et al., 2016). Despite ATP effects may be mediated by breakdown to adenosine, the negative chronotropic action of ATP was insensitive to blockage of adenosine A_1_ receptors with DPCPX used in a 6-fold higher concentration (3 nM) than that required to block this receptor (*K* *_i_*∼0.45 nM) (Lohse et al., 1987). However, one cannot exclude ATP conversion into adenosine during incubation with the nucleotide, yet even if this had occurred in our experimental conditions the amount of adenosine falls below the threshold to activate A_1_ receptors in the SAN. These findings contrast with those obtained by Camara et al. (2015); these authors concluded that the negative chronotropic effect of ATP was dependent on A_1_ receptors activation by using DPCPX in a concentration (1 µM) that is more than 2,000-fold higher than the *K* *_i_* value for this antagonist to block the A_1_ receptor (Camara et al., 2015). Under such conditions, off-target effects of DPCPX may appear, which include inhibition of phosphodiesterases that may explain reversal of the negative chronotropic effect of ATP (Camara et al., 2018).

Our theory that the negative chronotropic effect of ATP in spontaneously beating atria strips is mediated primarily *via* the activation of nucleotide-sensitive P2 purinoceptors is further supported by the fact that 1) it was reproduced by the enzymatically stable ATP analogue, ATPγS, 2) it was blocked by PPADS, a non-selective P2 purinoceptors antagonist exhibiting no affinity for adenosine receptors, and 3) prevention of ATP breakdown into adenosine with the NTPDase inhibitor, POM-1, increased rather than decreased ATP-induced negative chronotropism. Our findings agree with previous reports in the literature about the role of ATP and related adenine nucleotides on cardiac function (Versprille and van Duyn, 1966; Lundberg et al., 1984; Camara et al., 2015) and questions the most accepted hypothesis that the negative chronotropic action of ATP is most likely due to A_1_ receptors activation after its rapid conversion into adenosine (Pelleg and Belhassen, 2010). While this hypothesis neglected the pivotal role of P2 purinoceptors in the control of spontaneous activity of the SAN, it has been demonstrated that ATP was more potent than adenosine in reducing heart rate (Pelleg et al., 1985; Sharma and Klein, 1988), which was interpreted as being due to an additional vagal reflex of ATP *via* sensory P2X2 and/or P2X3 receptors (Pelleg et al., 1987; Xu et al., 2005). This idea is difficult to admit in the present experimental conditions due to the fact that blockage of muscarinic acetylcholine receptors with atropine (1µM) failed to affect the rate and tension of spontaneous atrial contractions and did not modify ATP-induced effects, thus indicating that the cholinergic vagal tone is irrelevant for the P2-mediated effects of the nucleotide.

To the best of our knowledge, this is the first study demonstrating a role for the P2X4 receptor in the regulation of sinoatrial node automatism. Despite limited availability of selective drugs acting on the P2X4 receptor, it may be pharmacologically characterized by comparing agonists rank order of potency: ATP > 2-methylthioATP > CTP > α, β-methyleneATP (Soto et al., 1996), as well as by the use of selective antagonists and allosteric modulators (reviewed in Stokes et al., 2017). The potent and selective P2X4 receptor antagonist, 5-BDBD, with an IC_50_ value of about 1 µM, attenuated the negative chronotropic effect of ATP on spontaneously beating atria, while the positive allosteric modulator of the P2X4 receptor, ivermectin, potentiated the nucleotide response. Furthermore, we show here that besides ATP and its stable analogue, ATPγS, also the P2X4 agonist, CTP, whose hydrolysis does not directly yield adenosine, decreased the spontaneous atrial rate in a 5-BDBD-sensitive manner, but with a weaker potency comparing with adenine nucleotides (Soto et al., 1996; Kasuya et al., 2017). In this study we used 5-BDBD at a concentration (10 µM) that might also interfere with P2X1- and P2X3-mediated actions (Coddou et al., 2019), but we are confident that this is irrelevant in this case because very low amounts of these receptors are expressed in the SAN (Musa et al., 2009).

The P2X4 receptor shares structural and functional properties with other P2X receptors. For instance, it is known that the rat P2X4 receptor is relatively insensitive to PPADS (IC_50_∼100 µM), in contrast to mouse and human P2X4 orthologs (IC_50_∼10 µM; Jones et al., 2000). Although exhibiting a weaker potency for the rat P2X4 receptor, we almost prevented the negative chronotropic effect of ATP using 100 µM PPADS. One cannot, however, exclude a minor participation of PPADS-sensitive metabotropic P2Y receptors in the bradycardic effect of ATP. The ionotropic P2X4 receptor is slowly desensitized by ATP (Jarvis and Khakh, 2009). This feature might explain the relatively sustained negative chronotropic effect of ATP and its analogue, ATPγS, during the time (at least for 5 min) of incubation with these compounds. However, the sustained negative chronotropic effect of ATP does not explain the potentiating action the NTPDase inhibitor, POM-1, unless one hypothesizes that extracellular ATP accumulation also contributes to reduce adenosine formation by feed-forwardly inhibiting ecto-5’-nucleotidase/CD73, as demonstrated in other studies (Magalhães-Cardoso et al., 2003; Duarte-Araújo et al., 2009; Vieira et al., 2014).

Notwithstanding our observations, other studies failed to demonstrate the involvement of P2X receptors in the control of heart rate. For instance, infusion of 2-methylthioATP did not change heart rate in the Langendorff-perfused heart (Mei and Liang, 2001), most probably because the used concentration (100 nM) of the ATP analogue falls below the threshold (1 µM) required to activate the P2X4 in the SAN (Jarvis and Khakh, 2009). The same group also failed to find any difference in the spontaneous heart rate when comparing wild-type with mice overexpressing or missing the P2X4 receptor (Hu et al., 2001; Yang et al., 2014). One must, however, emphasize that these studies were designed to evaluate the P2X4 receptor tone under basal conditions, *i.e.* in the absence of any P2X4 agonist, which is a different situation from the present report. Thus, future studies are required to elucidate the role of the P2X4 receptor in the *in vivo* control of heart rate.

In the rat heart, the P2X4 receptor is the third most abundant P2X receptor after P2X7 and P2X5 receptors, while in the human heart it is considered the most expressed P2X receptor subtype (Musa et al., 2009). Also the regional distribution of the P2X4 receptor in the heart displays some differences among species. Using immunofluorescence confocal microscopy, we show here that the P2X4 protein is slightly more expressed in the plasma membrane of SAN cells (mostly cardiomyocytes, but also blood vessels and nerve fibers) followed by the RV and RA of the rat. This is slightly different from data obtained in humans where the P2X4 receptor mRNA seems to be evenly expressed through the myocardium (Musa et al., 2009).

### P2X4-Induced Negative Chronotropism Requires Reversal of the NCX Activity Mode

Interestingly, the distribution of the P2X4 receptor in SAN cardiomyocytes matches the immunofluorescence staining pattern of NCX1 and HCN4 in the rat. This led us to hypothesize that the P2X4 receptor-mediated negative chronotropic effect of ATP could involve downstream modulation of NCX and/or HCN pacemaker activities. Crosstalk between P2X4- and NCX-mediated effects has been demonstrated (Shen et al., 2014). Opening of the P2X4 ion pore mediates the influx of positive charges, mainly Na^+^ and Ca^2+^ in a 1:4 ratio (Jarvis and Khakh, 2009), in the proximity of NCX carriers, which might affect their operation mode. Indeed, the ATP analogue, 2-methylthioATP (3 µM), inhibited the electrogenic forward mode of NCX in ventricular myocytes *via* an increase (by about 1 mM) in the intracellular Na^+^ concentration (Shen et al., 2014), which represents a net increase of 7–25% considering the resting intracellular Na^+^ concentration (Despa and Bers, 2013). Increases in intracellular Na^+^ may be even more relevant in cells with limited pathways for Na^+^ entry due to low expression levels of voltage-sensitive Na^+^ channels, like the SAN cardiomyocytes (Remme and Bezzina, 2010). Likewise, it has been demonstrated that persistent Na^+^ currents evoked by veratridine triggers intracellular calcium transients by reversing the operation mode of NCX in CA1 pyramidal cells (Fekete et al., 2009). Although speculative, reversal of the NCX function mode by the influx of Na^+^ represents an alternative mechanism for dysrhythmias (including bradycardia) in some inherited cardiac sodium channelopathies, such as the type 3 long QT syndrome associated with SCN5A mutations and persistent sodium currents (Remme and Bezzina, 2010). In agreement with our theory that Na^+^ influx *via* the P2X4 receptor pore might affect the NCX mode of function to decrease heart rate (**Figure 7**), we showed here for the first time that partial blockage of NCX, but not HCN channel, with two distinct inhibitors, KR-R7943 or ORM-10103, turned the spontaneously beating atria less sensitive to the negative chronotropic effect of ATP. Although beyond the scope of the present work, the interplay between P2X4 and NCX deserves further investigations using highly-demanding electrophysiology patch-clamp techniques in acutely isolated SAN cardiomyocytes from both rats and humans (ongoing research project).

Co-localization of P2X4 and NCX1 immunoreactivity in NF160 positive neuronal fibers was also detected. The presence of the P2X4 receptor in neuronal structures is widely accepted, but its function remains to be explored (Stokes et al., 2017). The SAN and the surrounding myocardium are regulated by a dense network of autonomic fibers, which are mainly parasympathetic followed by a sympathetic origin (Crick et al., 1994; Crick et al., 1999; Pauza et al., 2013; Zarzoso et al., 2013; Rajendran et al., 2019). Interestingly, some intracardiac neurons within atria contain ATP stored in vesicles (Crowe and Burnstock, 1982), which upon activation may represent an important source of extracellular ATP (Burnstock, 1972; Fredholm et al., 1982; Tokunaga et al., 1995). Reversion of NCX forward activity during ischemic conditions contributes to increase the magnitude of Ca^2+^ transients and, thus, the release of neurotransmitters from presynaptic nerve terminals (Lee and Kim, 2015). Given the co-localization and putative interplay between the P2X4 receptor and NCX in NF160-positive nerve fibers, one may speculate that these players may also interact to control the activity of cardiac neurons (Griffioen et al., 2007). This is even more relevant taking into consideration that cardiac ischemia is accompanied by P2X4 overexpression, particularly in the SAN (Musa et al., 2009). Thus, ATP released from autonomic cardiac nerves may trigger a positive feedback loop involving the NCX leading to an increase in the purinergic control of atrial cardiomyocyte function at both pre- and post-junctional levels.

### ATP-Induced Negative Inotropism Is Partially Offset by P2X4 Activation and NCX Transport Reversal

The inotropic effect of ATP was investigated in paced RV strips; the nucleotide decreased ventricular inotropy in a concentration-dependent manner, yet changes in paced ventricular tension were less potent than the recorded ATP-induced negative chronotropic actions in spontaneously beating atria. This raised the possibility for the existence of a yet unraveled ATP-induced negative inotropic offsetting mechanism. Although we did not fully characterized the receptors involved in the negative inotropic effect of ATP, previous studies agree that P2 purinoceptors activation may be necessary, also taking into consideration that adenosine plays a minor (if any) role on ventricular inotropy (Burnstock and Meghji, 1983; Belardinelli et al., 1995; Balogh et al., 2005). There is, however, a contention regarding to whether ATP exerts a positive or a negative inotropic effect on ventricular contractions. In contrast to our findings, most reports in the literature suggest that ATP exerts a predominant positive inotropic effect in the heart. Nonetheless, it is worth to emphasize that the vast majority of these studies were performed in isolated ventricular myocytes (Danziger et al., 1988; De Young and Scarpa, 1989; Christie et al., 1992; Podrasky et al., 1997; Mei and Liang, 2001; Balogh et al., 2005). These findings attenuate the theory that ATP-induced positive inotropism could be mediated by P2X4 receptors facilitating noradrenaline release from sympathetic nerve terminals in paced ventricular strips. Only three studies were performed in more complex tissue preparations, namely in rat papillary muscles (Legssyer et al., 1988; Scamps et al., 1990) and in the frog ventricle (Flitney and Singh, 1980). Interestingly, the Legssyer’s and Flitney’s studies reported a dual and opposing role of ATP in cardiac tissues. In support of a negative inotropic role for ATP, a recent study performed in intact isolated hearts, as well as in ventricular fragments and acutely isolated myocytes proposed that diadenosine tetraphosphate decreased ventricular inotropy probably *via* the activation of P2Y purinoceptors (Pakhomov et al., 2018).

Notwithstanding the conflicting results regarding the nature of the inotropic role of ATP, the use of ATP analogues and more selective P2 receptor modulators, in combination with genetic and other advanced biochemical techniques, provided strong evidence that several P2X and P2Y receptors may be positive ventricular inotropic mediators (reviewed in Erlinge and Burnstock, 2008; Burnstock and Pelleg, 2015). Regarding the P2Y receptor family, positive inotropy is generally attributed to stimulation of Gs and Gq-protein coupled receptors (Erlinge and Burnstock, 2008). Among them, ATP preferentially activates the P2Y_11_ receptor (Abbracchio et al., 2006). The selective P2Y_11_ agonist, AR-C67085, increased contraction in isolated cardiomyocytes as well as in isolated trabecular preparations. In that study, P2Y_12_ and P2Y_13_ receptors were excluded by the lack of effect of the stable ADP analogue, 2-methylthioADP, in cardiomyocytes contractile activity, which also nearly exclude any involvement of ATP-sensitive Gi-protein coupled P2Y receptor (Balogh et al., 2005). Of note, it is likely that these authors performed their studies in a mixed population of ventricular and atrial cardiomyocytes, as these cells were not separated by the enzymatic digestion of the heart. The putative involvement of P2Y receptors in the negative inotropic effect of ATP in paced ventricular strips was not assessed here, which is a limitation of our study that certainly deserves further investigations along with the corresponding effects in the *in vivo* animal.

Given the involvement of the P2X4 receptor in the negative control of sinoatrial automatism (see above), we focused our interest at investigating the role of this ionotropic receptor on ventricular contractile activity (Erlinge and Burnstock, 2008; Burnstock and Pelleg, 2015). This question was raised because heart rate slowing drugs devoid of effect or with a moderate positive inotropic action on ventricular contraction may be relevant to treat heart failure. Our findings show that selective blockage of the P2X4 receptor activation with 5-BDBD significantly increased the negative inotropic effect of ATP in paced RV strips, thus suggesting that the P2X4 receptor may exert a counteracting positive inotropic action that is responsible for partially offsetting ATP-induce downsizing of ventricular contractions. As a matter of fact, the ATP analogues, 2-methylthioATP and α,β-methyleneATP, increased contractions of isolated ventricular cells, as well as of ventricular strips and isolated working hearts in rodents (Burnstock and Meghji, 1983; Hu et al., 2001; Mei and Liang, 2001). Overexpression of the P2X4 receptor 1) enhances ATP-induced cardiac contractility in the intact heart, and 2) rescues the systolic function and increase survival of animals with cardiomyopathy (Yang et al., 2004; Shen et al., 2009). The beneficial effects of the P2X4 receptor on cardiac function were attributed to activation of calcium-dependent endothelial-type nitric oxide synthase (Blaustein and Lederer, 1999; Yang et al., 2015). Ca^2+^ influx through the P2X4 receptor pore may itself account for the positive inotropic action of ATP analogues. On the other hand, Na^+^ influx through the P2X4 receptor also contributes to inhibit or, even revert, the electrogenic transport of NCX in the forward mode (Ca^2+^ extrusion mode) leading to an additional increase in the amplitude and duration of Ca^2+^ transients inside cardiomyocytes, which boosts their contractile activity (Shen et al., 2014; see **Figure 7**).

This concept may also explain the biphasic effect of ATP on atrial inotropy reported in this study and by other authors (Froldi et al., 1994; Gergs et al., 2008), which consisted of a transient decrease followed by a gradual recovery of the amplitude of atrial contractions while the preparations were still in contact with the nucleotide. Even though atrial inotropy represents an important reserve to maintain cardiac output in demanding conditions and in the setting of ventricular diastolic dysfunction, this phenomenon was not further evaluated in spontaneously beating rat atria due to significant bias introduced by changes in the rate of contractions.

Another relevant aspect of the P2X4 receptor regulation with potential implications for cardiac pathophysiology is its sensitivity to pH; in acidotic conditions, as it occurs in ischemia/hypoxia or renal failure, the P2X4 receptor activity significantly decreases, whereas the opposite occurs alkaline conditions (Wildman et al., 1999). Growing evidence exist demonstrating that the P2X4 receptor is overexpressed in ventricles under stressful conditions, namely in pulmonary hypertension and ischemia-induced heart failure (Sonin et al., 2008; Musa et al., 2009; Ohata et al., 2011). Altogether these findings strengthen the potential involvement of the P2X4 receptor in cardiac normal physiology and diseases progression.

## Conclusion

Overall, data suggest that ATP-sensitive P2X4 ionotropic receptors play a major role in decreasing the spontaneous activity of the SAN while partially offsetting the negative inotropic effect of the nucleotide in paced rat ventricles. The mechanism underlying the dual P2X4 receptor-mediated effects on cardiac chronotropy and inotropy involves downstream interaction with the activity of NCX. Na^+^ influx *via* the P2X4 receptor pore may inhibit and/or revert the electrogenic forward current of the NCX, thus decreasing chronotropy. Likewise, intracellular Ca^2+^ accumulation due to interference with NCX might explain the positive inotropic effect attributed to the P2X4 receptor activation on paced RV strips (**Figure 7**). Regional differences observed for the distribution of the P2X4 receptor, along with its biophysical properties, bring new therapeutic opportunities for P2X4 activation with potential to create novel well-tolerated heart-rate lowering drugs with promising benefits in patients with deteriorated ventricular function.

## Data Availability

All datasets generated for this study are included in the manuscript/**Supplementary Files**.

## Ethics Statement

The animal study was revised and approved by the competent national authority Direção Geral de Alimentação e Veterinária, and by the ICBAS Animal Ethical Committee (No. 224/2017).

## Author Contributions

PC-S supervised the project. BB and PC-S designed the experiments and wrote the manuscript. BB and SN-M carried out myographic recordings. BB and FF performed immunofluorescence confocal microscopy experiments. BB, FF, AF-S, and PC-S interpreted data, discussed the clinical implications, and commented on the manuscript at all stages.

## Funding

This work was supported by Foundation for Science and Technology (FCT) (FCOMP-01-0124-FEDER-028726-FEDER, COMPETE-FCT PTDC/DTP-FTO/0802/2012, PEst-OE/SAU/UI0215/2014, UID/BIM/4308/2016 and UID/BIM/4308/2019). The funders had no role in study design, data collection and analysis, decision to publish, or preparation of the manuscript. BB is in receipt of a PhD studentship from FCT (FEDER funding SFRH/BD/104114/2014).

## Conflict of Interest Statement

The authors declare that the research was conducted in the absence of any commercial or financial relationships that could be construed as a potential conflict of interest.

## Supplementary Material

Figure S1 ATP (0.1 µM–1 mM) concentration-dependently decrease the spontaneous atrial rate. The negative chronotropic effect of ATP was mimicked by its enzymatically stable analogue, ATPγS (0.001–0.1 mM, A), and its potency was increased after pretreatment of the preparations with POM-1 (100 µM, a non-selective NTPDase inhibitor, B) and with ivermectin (30 µM, a positive allosteric modulator of the P2X4 receptor, C). For comparison purposes, we show in panel D that ATP (100 µM)-induced negative chronotropy was fully blocked by PPADS (100 µM). In panels A–C, data are expressed as mean ± SEM from an *n* number of animals. In panel D, represented are box-and-whiskers plots, with whiskers ranging from minimum to maximum values calculated as a percentage (%) of variation from baseline; horizontal lines inside boxes indicate the corresponding medians; each data point represents the result of a single experiment; data from the same experiment are connected by lines. *p < 0.05 (Student’s *t*-test for paired samples) represent significant differences when compared to the effect of ATP alone.

Figure S2 Effects of receptor antagonists/allosteric modulators, NTPDase inhibitor and ion transport blockers on spontaneously beating rat atria (A–J). POM-1 (100 µM, A), PPADS (10 µM, B; 100 µM, C), DPCPX (3 nM, D), A438079 (3 µM, E), 5-BDBD (10 µM, F), ivermectin (30 µM, G), KB-R7943 (3 µM, H), ORM-10103 (3 µM, I), and ZD7288 (300nM, J) contacted with the preparations at least for 15 min; the rate of spontaneous atrial contractions (beats min^−1^) was measured immediately before ATP applications (see Figures 2–4) and compared to baseline conditions in the absence of any drug. Each data point represents the result of a single experiment; points from the same experiment are connected by lines. On the right hand-side of each panel, represented are box-and-whiskers plots, with whiskers ranging from minimum to maximum values calculated as a percentage (%) of variation from baseline; horizontal lines inside boxes indicate the corresponding medians. *p < 0.05 (Student’s *t*-test for paired samples) represent significant differences when compared to baseline.

Figure S3 Effects of the P2X4 receptor antagonist, 5-BDBD (10 µM, A and B) and of the NCX inhibitor, KB-R7943 (3 µM, C and D) on paced right ventricular contractions. Drugs contacted with the preparations at least for 15 min; the amplitude of 2 Hz-paced ventricular contractions measured as active tension (mN/mg of wet tissue weight, panels A and C) and as the derivative of developed force over time (+dF/dt, mN/s, panels B and D) was measured immediately before ATP applications (see **Figure 5**) and compared to baseline conditions in the absence of any drug. Each data point represents the result of a single experiment; points from the same experiment are connected by lines. On the right hand-side of each panel, represented are box-and-whiskers plots, with whiskers ranging from minimum to maximum values calculated as a percentage (%) of variation from baseline; horizontal lines inside boxes indicate the corresponding medians. *p < 0.05 (Student’s *t*-test for paired samples) represent significant differences when compared to baseline.

Figure S4 Representative confocal micrographs showing the immunolocalization of the P2X4 receptor (Apr-024, extracellular loop, Alomone) and of the HCN4 channel (Agp-004, Alomone) in the rat sinoatrial node (SAN); images obtained in right atria (RA) and right ventricle (RV) are also shown for comparison. It is worth noting that while the SAN was positive for both markers, RA and RV were positive for the P2X4 receptor (green) but negative for the HCN4 (magenta). Images were taken from one whole-mount preparation of the rat heart including the three regions, SAN, RA and RV. Dashed lines represent boundaries of the SAN region. White arrows indicate blood vessels including the SAN artery. Scale bar 30 µm.

Figure S5 Representative confocal micrographs of SAN showing positive immunoreactivity against P2X4 (Apr-002, Alomone), NCX1 (Anx-011, Alomone), and NF160 (Ab7794, Abcam) proteins. The upper three panels show lower magnification confocal images of the SAN identified by the enrichment in NF160-positive nerve fibers; images also show surrounding atrial cardiomyocytes characterized as being NF160-negative and Cx43-positive. Images were taken from only one atrial preparation. Dashed lines represent boundaries of the SAN region. White arrows indicate blood vessels including the SAN artery. Scale bar 30 µm.
