# Supplementary figures and images for: The Ionotropic P2X4 Receptor has Unique Properties in the Heart by Mediating the Negative Chronotropic Effect of ATP While Increasing the Ventricular Inotropy
Source: Front Pharmacol. 2019 Sep 24;10:1103. doi: 10.3389/fphar.2019.01103 (PMC6769074; doi:10.3389/fphar.2019.01103)

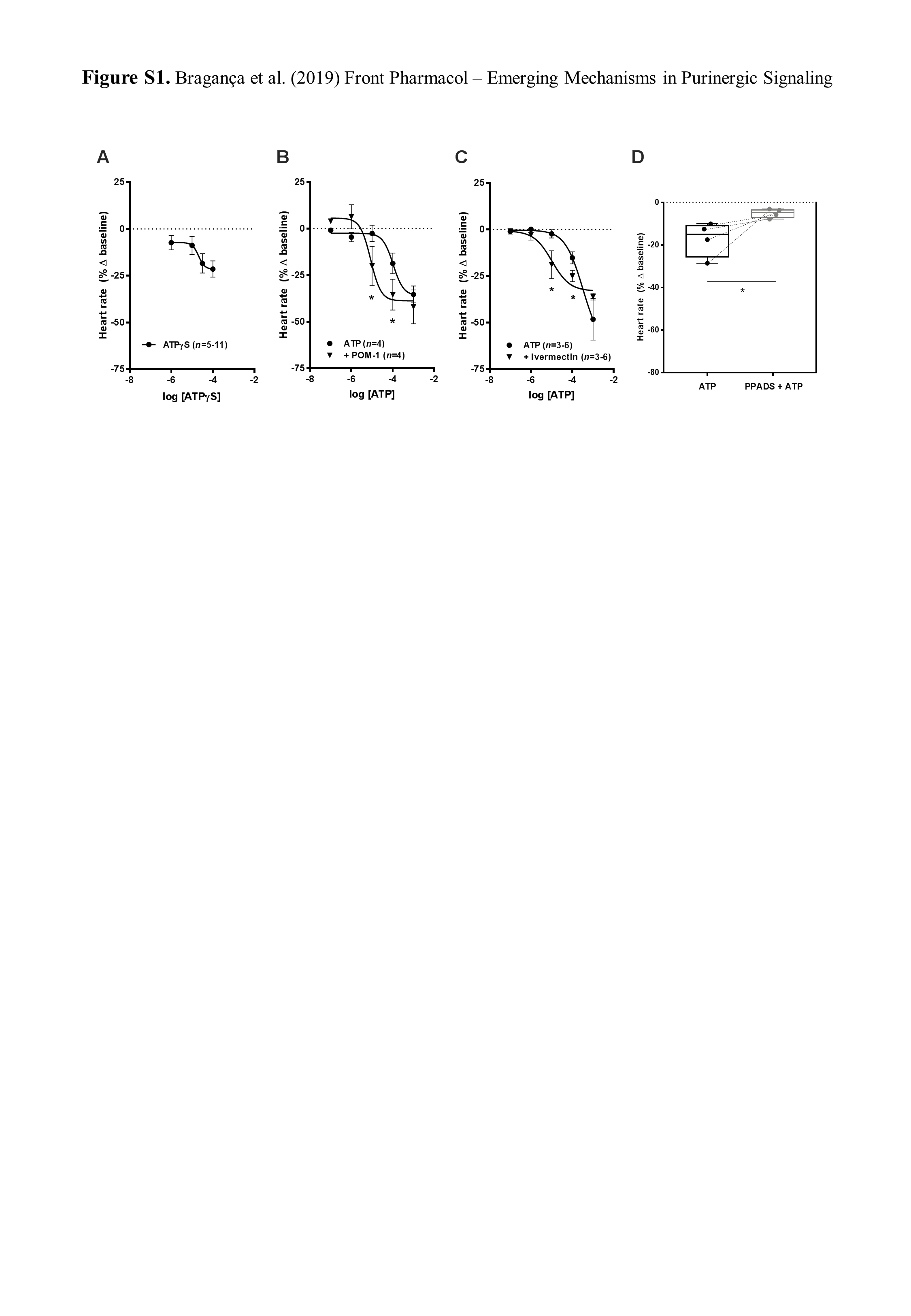

Supplement: Figure S1 — ATP (0.1 µM–1 mM) concentration-dependently decrease the spontaneous atrial rate. The negative chronotropic effect of ATP was mimicked by its enzymatically stable analogue, ATPγS (0.001–0.1 mM, A), and its potency was increased after pretreatment of the preparations with POM-1 (100 µM, a non-selective NTPDase inhibitor, B) and with ivermectin (30 µM, a positive allosteric modulator of the P2X4 receptor, C). For comparison purposes, we show in panel D that ATP (100 µM)-induced negative chronotropy was fully blocked by PPADS (100 µM). In panels A–C, data are expressed as mean ± SEM from an n number of animals. In panel D, represented are box-and-whiskers plots, with whiskers ranging from minimum to maximum values calculated as a percentage (%) of variation from baseline; horizontal lines inside boxes indicate the corresponding medians; each data point represents the result of a single experiment; data from the same experiment are connected by lines. *p < 0.05 (Student’s t-test for paired samples) represent significant differences when compared to the effect of ATP alone. [file Image_1.tif]

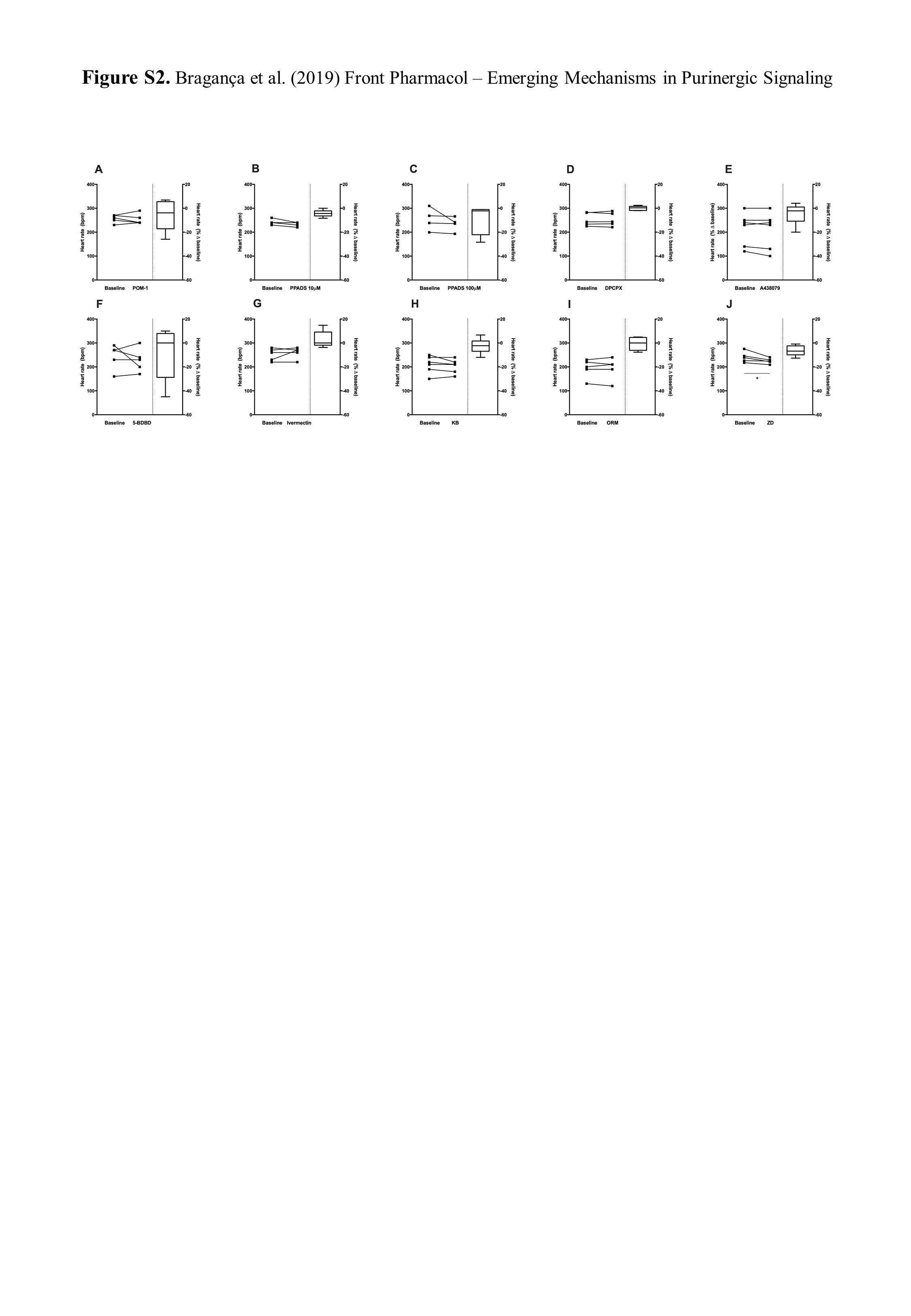

Supplement: Figure S2 — Effects of receptor antagonists/allosteric modulators, NTPDase inhibitor and ion transport blockers on spontaneously beating rat atria (A–J). POM-1 (100 µM, A), PPADS (10 µM, B; 100 µM, C), DPCPX (3 nM, D), A438079 (3 µM, E), 5-BDBD (10 µM, F), ivermectin (30 µM, G), KB-R7943 (3 µM, H), ORM-10103 (3 µM, I), and ZD7288 (300nM, J) contacted with the preparations at least for 15 min; the rate of spontaneous atrial contractions (beats min−1) was measured immediately before ATP applications (see Figures 2–4) and compared to baseline conditions in the absence of any drug. Each data point represents the result of a single experiment; points from the same experiment are connected by lines. On the right hand-side of each panel, represented are box-and-whiskers plots, with whiskers ranging from minimum to maximum values calculated as a percentage (%) of variation from baseline; horizontal lines inside boxes indicate the corresponding medians. *p < 0.05 (Student’s t-test for paired samples) represent significant differences when compared to baseline. [file Image_2.tif]

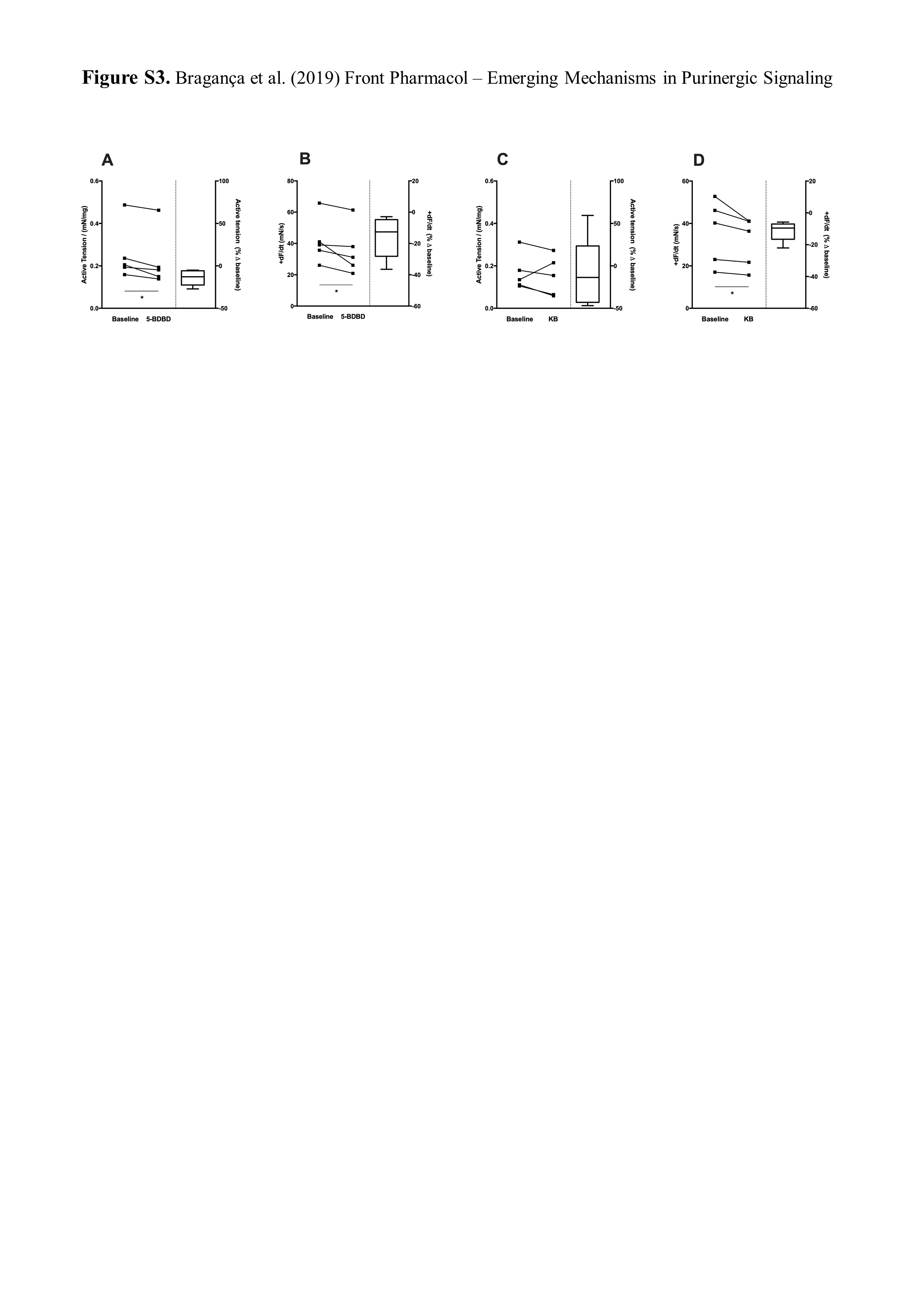

Supplement: Figure S3 — Effects of the P2X4 receptor antagonist, 5-BDBD (10 µM, A and B) and of the NCX inhibitor, KB-R7943 (3 µM, C and D) on paced right ventricular contractions. Drugs contacted with the preparations at least for 15 min; the amplitude of 2 Hz-paced ventricular contractions measured as active tension (mN/mg of wet tissue weight, panels A and C) and as the derivative of developed force over time (+dF/dt, mN/s, panels B and D) was measured immediately before ATP applications (see Figure 5 ) and compared to baseline conditions in the absence of any drug. Each data point represents the result of a single experiment; points from the same experiment are connected by lines. On the right hand-side of each panel, represented are box-and-whiskers plots, with whiskers ranging from minimum to maximum values calculated as a percentage (%) of variation from baseline; horizontal lines inside boxes indicate the corresponding medians. *p < 0.05 (Student’s t-test for paired samples) represent significant differences when compared to baseline. [file Image_3.tif]

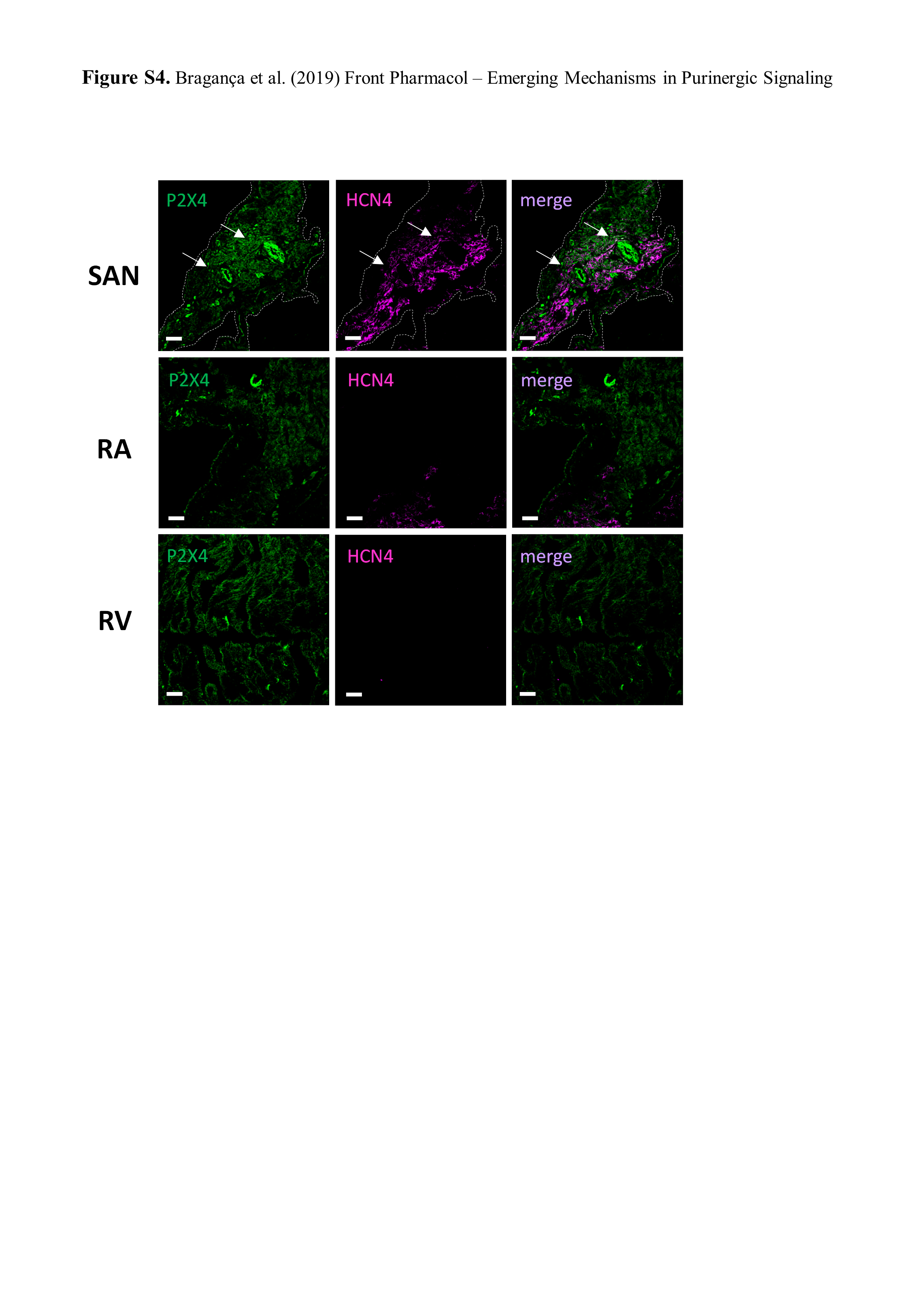

Supplement: Figure S4 — Representative confocal micrographs showing the immunolocalization of the P2X4 receptor (Apr-024, extracellular loop, Alomone) and of the HCN4 channel (Agp-004, Alomone) in the rat sinoatrial node (SAN); images obtained in right atria (RA) and right ventricle (RV) are also shown for comparison. It is worth noting that while the SAN was positive for both markers, RA and RV were positive for the P2X4 receptor (green) but negative for the HCN4 (magenta). Images were taken from one whole-mount preparation of the rat heart including the three regions, SAN, RA and RV. Dashed lines represent boundaries of the SAN region. White arrows indicate blood vessels including the SAN artery. Scale bar 30 µm. [file Image_4.tif]

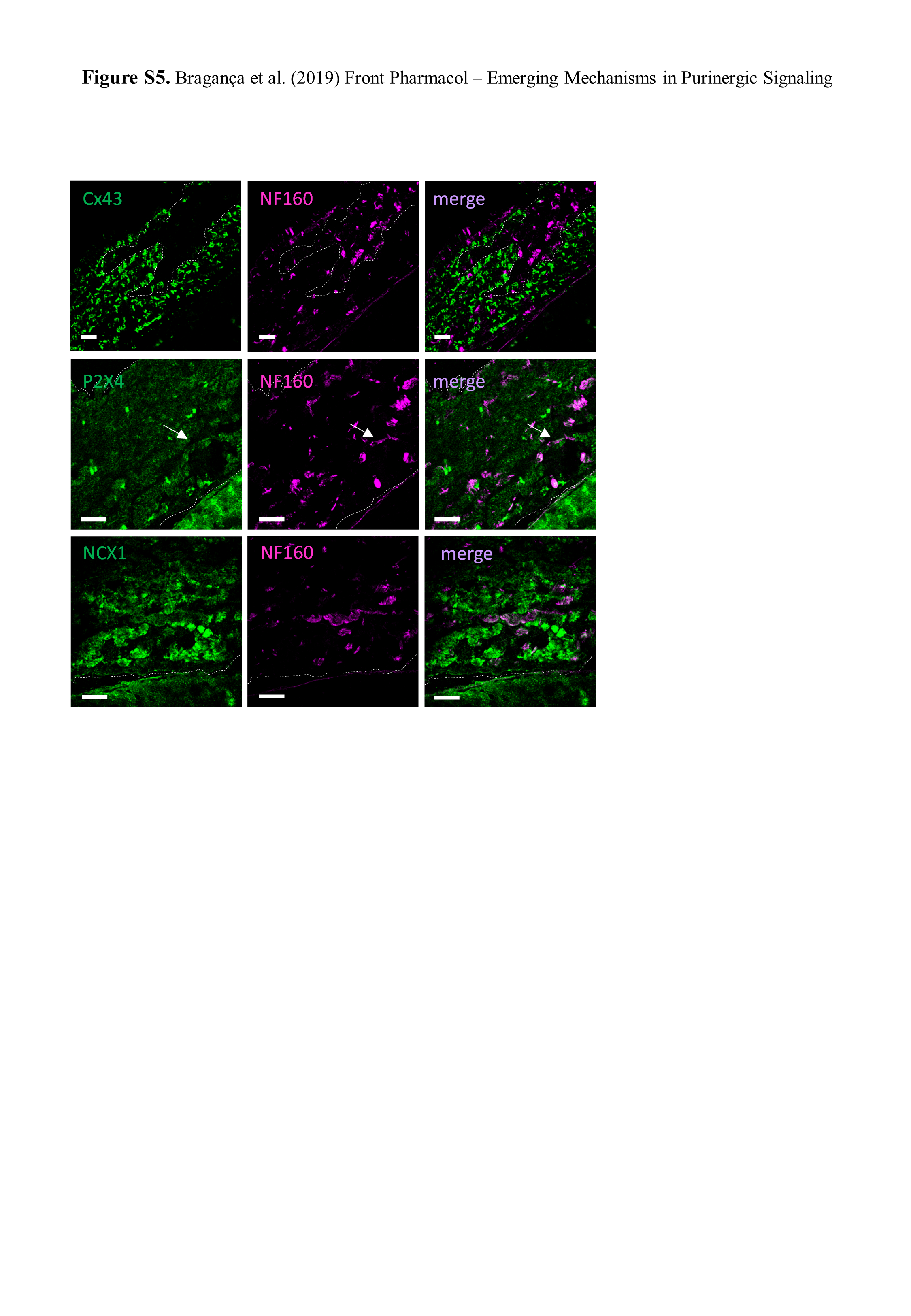

Supplement: Figure S5 — Representative confocal micrographs of SAN showing positive immunoreactivity against P2X4 (Apr-002, Alomone), NCX1 (Anx-011, Alomone), and NF160 (Ab7794, Abcam) proteins. The upper three panels show lower magnification confocal images of the SAN identified by the enrichment in NF160-positive nerve fibers; images also show surrounding atrial cardiomyocytes characterized as being NF160-negative and Cx43-positive. Images were taken from only one atrial preparation. Dashed lines represent boundaries of the SAN region. White arrows indicate blood vessels including the SAN artery. Scale bar 30 µm. [file Image_5.tif]
